# Fresh Record of Family Cyprinidae From River Kurram at Bannu, Khyber Pakhtunkhwa, Pakistan: A Statistical Analysis

**DOI:** 10.1155/sci5/2603978

**Published:** 2025-09-07

**Authors:** Abdul Haseeb, Ali Muhammad Yousafzai, Latif Ahmad, Muhammad Ismail Khan, Umair Khan, Syed Modassir Hussain

**Affiliations:** ^1^Department of Zoology, Islamia College University Peshawar, Peshawar, Pakistan; ^2^Department of Mathematics, Shaheed Benazir Bhutto University, Sheringal 18000, Pakistan; ^3^Department of Computer Science and Mathematics, Lebanese American University, Byblos 1401, Lebanon; ^4^Department of Mathematics, Faculty of Science, Sakarya University, Sakarya 54050, Turkey; ^5^Department of Mathematics, Faculty of Science, Islamic University of Madinah, Madinah 42351, Saudi Arabia

**Keywords:** Barbinae, Cyprinidae, Cyprininae, diversity, ichthyofauna, River Kurram

## Abstract

The present study explores the ichthyodiversity of a Cyprinid fish. A total of 622 fish samples were collected from five different sites of the Kurram River and identified using standard keys for identification. Out of 622 collected fish samples, 188 were related to the family Cyprinidae comprised of 19 species such as *Barilius vagra, Barilius modestus, Barilius pakistanicus, Cyprinus carpio, Labeo rohita, Carassius auratus, Salmophasia punjabensis, Amblypharyngodon mola, Gara gotyla, Puntius conchonius, Puntius chola, Puntius sophore, Puntius ticto, Tor putitora, Schizothorax esocinus*, *Schizothorax plagiostomus*, *Schizothorax labiatus, Labeo diplocheilus*, and *Crossocheilus diplocheilus.* The current study explores 5 new species, i.e., *Schizothorax labiatus, Schizothorax esocinus, Amblypharyngodon mola, Puntius chola*, and *Salmophasia punjabensis* for the first time, which are not reported in the earlier work. Principal component analysis (PCA) and diversity indices were analyzed using XLSTAT in conjunction with Microsoft Excel 2019 to assess the correlation and richness of fish diversity. To check the water quality of the River Kurram, the following parameters were examined: temperature, copper, pH, nitrate, alkalinity, chlorine, total hardness, iron, nitrite, and lead. All the recorded physicochemical parameters remained within the safe limits throughout the study period.

## 1. Introduction

**Figure 1:**
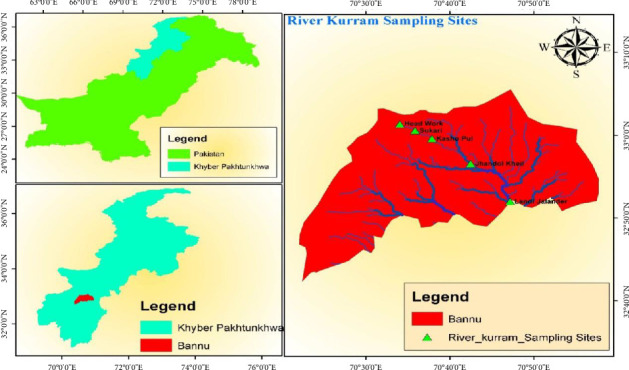
Different collection sites of the River Kurram.

The Kurram River flows into Kurram Agency, Pakistan, from the Afghan Provinces of Paktia and Khost, and drains into the Spin Ghar mountain range (Figure 1). The River Kurram rises roughly 20 km to the southeast of Gardez in the Afghan Province of Paktia. It passes through the Northeastern Mountains before entering Pakistan around 80 km to the southwest of Jalalabad. The river originates from the southern slopes of the Koh-e-Sufed Mountains, flows at the north of Bannu, and after covering a distance of about 330 km, it joins the River Indus near Essa Khel, where the plains are reached [1].

Fourteen thousand of the 35,100 species of fish included in FishBase are related to freshwater [2]. Fish plays a critical part in the growth of a country and is an inexpensive, highly nutritious source of protein in addition to supplying vital vitamins and minerals that the human body requires [3].

Agnatha, Chondricthyes, and Osteichthyes are the three primary super classes into which class Pisces has been divided. Osteichthyes has been divided into several orders and families, one of which being the family Cyprinidae, much like other super classes [4]. With 220 genera and 2400 species, the family Cyprinidae is regarded as the largest [5]. Cyprinids are considered as very essential food fish globally. Furthermore, a lot of cyprinid species, such as goldfish, are now commonly housed in ponds and aquariums as ornamental fish [6].

Worldwide and in countries such as Japan, China, Bangladesh, India, and Pakistan, aquaculture uses a variety of cyprinids, such as Labeo rohita, Cyprinus carpio, Catla catla, and Cirrhinus mrigala [7].

Ten of the 21 freshwater fish species identified by Owais and Uma [8], during their investigation of Sagar Lake, India, belonged to the Cyprinidae family. Tesia and Bordoloi [9] report that 16 species of the Cyprinidae family have been found in Arunachal Pradesh, India's Charju River. Thirty species of freshwater fish were gathered from the Tamor River in the Himalayan region of Nepal. Cyprinidae accounted for 61 percent of the fish [10]. 29 of the 66 fish species recorded in the Similipal Biosphere Reserve came from the family Cyprinidae, according to a research [11].

Pakistan has 86 species of freshwater fish, according to Rafique and Khan [12]. According to Khattak et al. [13], there are 14 species of the Cyprinidae family known to exist in the River Kabul in the District Nowshera. From the Jhelum River, 51 different species of fish were gathered, 25 fish of these were connected to the Cyprinidae family [1]. From the River Zhob in Baluchistan, six species of the Cyprinidae family were recovered [14]. From the Swat River near Charsadda, 20 species of Cyprinidae have been identified [15]. In a study, Wahab and Yousafzai [16] found 10 species of cyprinids in the River Panjkora Dir Lower.

Of the 25 fish species found in the River Kurram, 16 are related to the family Cyprinidae, according to Rehman et al. [17]. Five of the eight species from the River Kurram that Masood et al. [18] reported were related to cyprinids. Three species of fish belonging to the family Cyprinidae were among the five fish that Ilyaset al. [19] collected and identified during their study of the freshwater fauna of the Damai stream in District Bannu.

Water quality emerges as a defining factor shaping the ichthyofaunal diversity of the Kurram River at Bannu, Khan et al. [20]. As the river flows from its pristine headwaters to the downstream sections, its water quality undergoes considerable changes due to agricultural runoff, urban pollution, and industrial discharges [21]. These changes in water quality can have profound implications for fish populations, affecting their health, reproductive success, and overall survival [20].

The purpose of applying principal component analysis (PCA) in fisheries research is to reduce the complexity of multivariate data such as environmental parameters, biological traits, and catch statistics into a smaller set of principal components that retain most of the original variability. This enables researchers to identify key factors influencing fish stock dynamics, habitat preferences, seasonal patterns, and the impact of environmental changes. PCA helps highlight relationships among variables, distinguish patterns across locations or time periods, and support data-driven decisions in sustainable fisheries management [6].

Our province has an abundance of natural water resources. Several major rivers, including the Panjkora, Kurram, Swat, and Kabul Rivers, combine to form the Indus River. Understanding and conserving this biodiversity hotspot requires a multifaceted approach that integrates scientific research, community engagement, and policy interventions [22]. By unraveling the mysteries of the Kurram River's ichthyofaunal diversity, we can unlock valuable insights into the ecological dynamics of riverine ecosystems and chart a course towards their sustainable management and conservation for generations to come [23]. Therefore, the main objective of this study was to find out the Cyprinidae family's present situation, distribution, and ichthyofauna in the River Kurram in Bannu, Khyber Pakhtunkhwa.

## 2. Materials and Methods

### 2.1. Study Area

The Kurram River originates in Afghanistan's Paktia Province, specifically from Kohe-e-Sufaid (also known as Speen Ghar), and flows into Pakistan's Kurram District previously called Kurram Agency, before passing through Bannu in the northwest and continuing rapidly southward into Lakki Marwat [24]. Its principal sources of water include runoff from nearby terrains, summer monsoon rainfall, and winter snowmelt [17]. This river greatly supports the farming operations in the Lower Kurram, Bannu, and Lakki Marwat regions by providing essential irrigation for agricultural lands [25]. The river's breadth, depth, and flow rate fluctuate along its roughly 240 km journey. As the river moves downstream, the amount of water released tends to decrease. Depending on height, winter temperatures can fall below freezing, while summer temperatures range from a comfortable 17°C–31°C [26].

### 2.2. Sample Collection

With the help of local fishermen, fish specimens were collected every two months from five different locations such as Head Works, Sukari, Jhando Kheil, Kashu Pul, and Shamshi Kheil along the Kurram River between March 2021 and September 2021. A range of fishing techniques was used, including simple hooks, rods, and various net types, such as hand and cast nets.

### 2.3. Morphological Identification

The collected samples were shifted to the laboratory of the zoology department at Islamia College Peshawar for morphological identification with the help of different standard fish identification keys [27]. External morphological characteristics, including body shape, mouth position, fin position, fin ray number and type, lateral line scale types and numbers, barbel length and position, body color pattern, and measurement of various morphometric variables, were used to identify the specimens.

### 2.4. Water Sample Collection and Analysis

Water samples were gathered randomly from several locations and kept in clean containers, that is, sterile bottles made of plastic. Digital thermometers and pH meters were used on the spot to measure temperature and pH, respectively. Samples that had been preserved were taken to the PCSIR lab in Peshawar for analysis of copper, alkalinity, chlorine, lead, total hardness, nitrite, iron, and nitrate.

### 2.5. PCA

PCA is a linear combination of many elements used to standardize factors for comparative analysis. It is then utilized to identify the variables that affect the sample and provides a definitive justification for the most important element. This publication used Excel 2019 and XLSTAT for many of the studies.

The map (Figure 1) was created by using ArcGIS software.

## 3. Results

**Figure 2:**
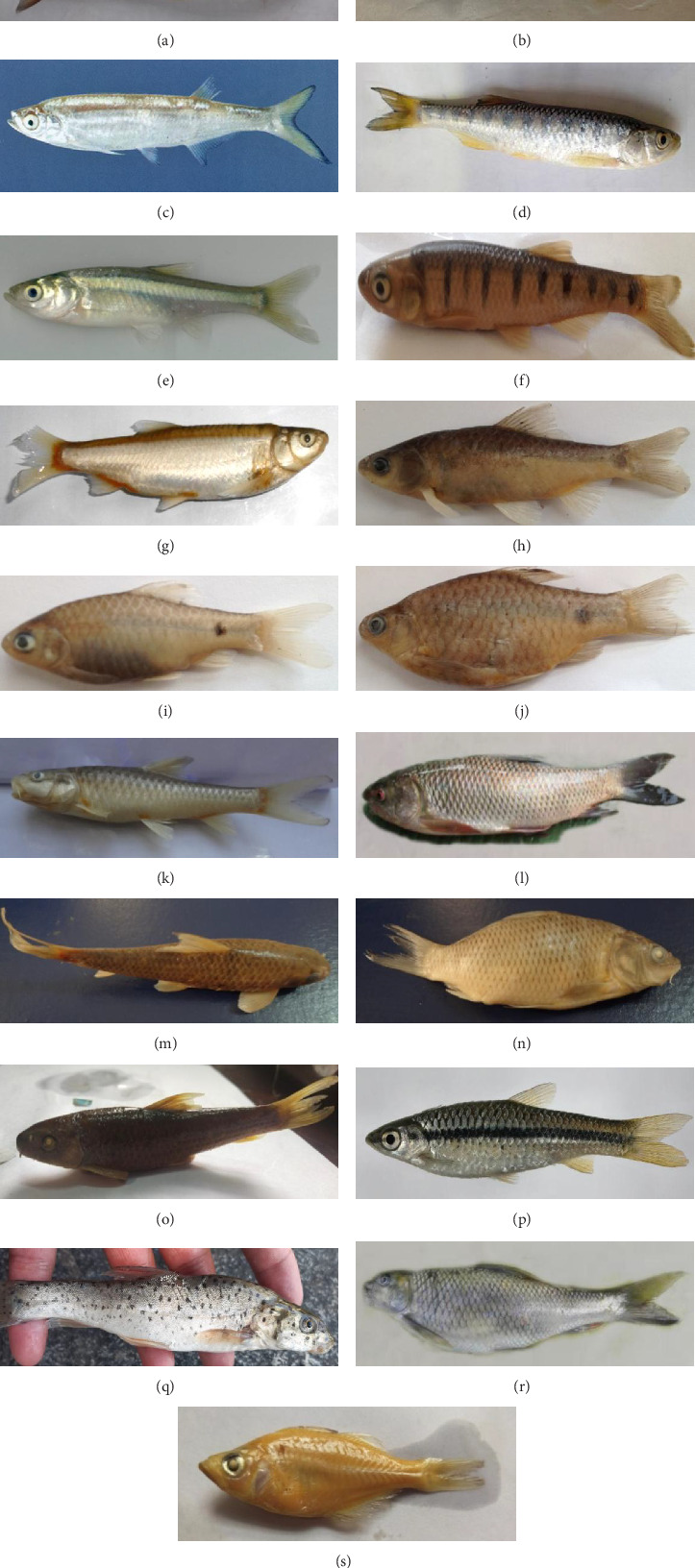
Fish diversity of cyprinids collected from the River Kurram Bannu, KP. (a) *Schizothorax labiatus*. (b). *Schizothorax plagiostomus*. (c). *Salmophasia punjabensis*. (d). *Barilius vagra*. (e). *Amblypharyngodon mola*. (f). *Barilius pakistanicus*. (g). *Barilius modestus*. (h). *Puntius sophore*. (i). *Puntius conchonius*. (j). *Puntius ticto*. (k). *Tor putitora*. (l). *Labeo rohita*. (m). *Garra gotyla*. (n). *Cyprinus carpio*. (o). *Crossocheilus diplocheilus*. (p). *Rasbora daniconius*. (q). *Labeo diplocheilus*. (r). *Schizothorax esocinus*. (s). *Carassius auratus*.

From all the sites that were chosen, a total of 19 different species of fish were collected. The Cyprininae represented two species, namely, *Cyprinus carpio* and *Carassius auratus*; the Danioninae represented five species, namely, *Barilius vagra, Barilius modestus, Barilius pakistanicus, Salmophasia punjabensis*, and *Amblypharyngodon mola*; the Labeoninae represented five species, namely, *Crossocheilus diplocheilus, Tor putitora, Labeo rohita, Gara gotyla,* and *Labeo diplocheilus*; and the Barbinae represented seven species, namely, *Puntius sophore, Puntius ticto, Puntius chola, Schizothorax esocinus, Puntius conchonius, Schizothorax plagiostomus,* and *Schizothorax labiatus* (Table 1 and Figures 2(a), 2(b), 2(c), 2(d), 2(e), 2(f), 2(g), 2(h), 2(i), 2(j), 2(k), 2(l), 2(m), 2(n), 2(o), 2(p), 2(q), 2(r), and 2(s)). Four subfamilies of the family Cyprinidae were represented: two species in the subfamily Cyprininae, five in the subfamily Danioninae, five in the subfamily Labeoninae, and seven in the subfamily Barbinae. With a relative frequency of 1.03 and a relative density of 19.6, *Schizothorax plagiostomus* was the most common species. *Amblypharyngodon mola* came in second with a relative frequency of 1.03 and a relative density of 6.38. When comparing Jhando Kheil with the other collecting sites, 41, 40, 38, 37, and 32, the species richness was higher (Table 2). Morphometric parameters of the collected cyprinids were observed and tabulated (Table 3). The following rare species were found in the study: *Puntius conchonius, Puntius sophore, Cyprinus watsoni, Puntius ticto*, and *Schizothorax esocinus*. The presence or absence of some species at different collecting locations is due to the variation in habitat, water depth, water quality, anthropogenic activities, and pollution. With seven species, the subfamily Barbinae became the most prevalent group. A study and summary of the physiochemical parameters of water collected from the River Kurram have been conducted and tabulated (Table 4).

Diversity indices were displayed and tallied, including dominance, individuals, Shannon, Simpson, evenness, and Margalef (Table 5).

**Figure 3:**
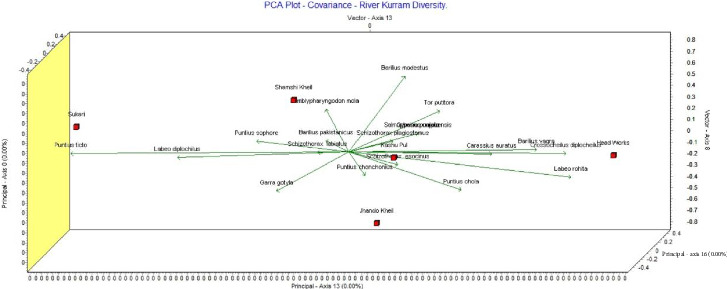
3D PCA plots of different cyprinids collected from different collection points of the Kurram River.

**Figure 4:**
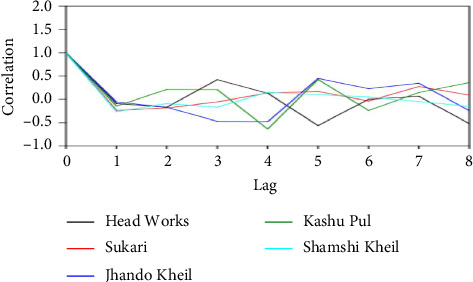
Correlation of cyprinids at the different collecting sites of the River Kurram.

**Figure 5:**
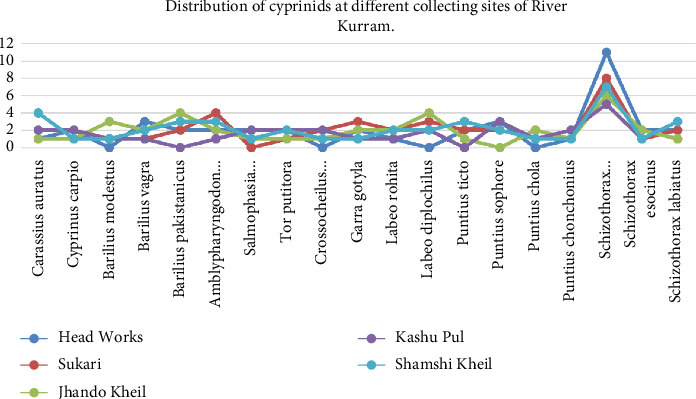
Distribution of cyprinids at the different collecting sites of the River Kurram.

Based on PCA, Shamshi Kheil is strongly associated with *Amblypharyngodon mola*. Jhando Kheil and Kashu Pul are closely associated with *Puntius chola, Schizothorax esocinus,* and *Puntius conchonius.* Head Works aligns with *Carassius auratus, Barilius vagra,* and *Crossocheilus diplocheilus.* Sukari is characterized by the presence of *Puntius ticto* and *Labeo diplocheilus*, suggesting these species are dominant or unique there. The factor loadings (FLs) may be classified into weak, moderate, or strong as illustrated in Figure 3. If FL is more than 0.75, it is deemed strong; if FL is between 0.75 and 0.50, it is deemed moderate. FL is regarded as weak if it is between 0.50 and 0.30 [28]. A correlation analysis was conducted between several collection locations, revealing that the majority of the data were correlated, indicating a correlation between their fish diversity (Table 6 and Figure 4). The FLs for the diversity of Cyprinidae are shown in Table 6, demonstrating the excellent contributions made by all the sites to this study (Figure 5).

## 4. Discussion

The current study's findings are consistent with previous research on the River Kurram conducted by Ur Rehman et al. [29]. That research identified 25 species of fish from the River Kurram, 16 of which are members of the Cyprinidae family, and include *Barilius modestus, Puntius conchonius, Barilius vagra, Barilius naseeri, Barilius pakistanicus, Puntius sophore, Puntius ticto, Cirrhinus reba, Cirrhinus mrigala, Labeo dyocheilus, Aspidoparia morar, Labeo boga, Crossocheilus diplocheilus, Systomus sarana, Chela*, and *Garra gotyla.* Eight of these species, such as*, Puntius conchonius, Barilius naseeri, Cirrhinus reba, Cirrhinus mrigala, Aspidoparia morar, Labeo boga, Systomus sarana*, and *Chela*, have not been reported in this study, and their absence may be due to differences in time and site of collection or climatic changes.

Ilyas et al. [19] found eight species from the River Kurram, of which five species *Labeo calbasu, Cirrhinus mrigala, Tor putitora, Labeo dyocheilus*, and *Barilius modestus* represented the Cyprinidae family, which was the most abundant. Of these five species, three are included in our analysis; the other two, *Labeo calbasu* and *Cirrhinus mrigala*, are absent in this investigation, which may be due to collection time or site difference.

Ilyas et al. [19] explored the fish fauna of the Damai stream in Bannu District. They collected and identified 3 species such as *Labeo rohita, Puntius sarana*, and *Barilius vagra*, which were connected to the Cyprinidae family. Of these, two, *Barilius vagra and Labeo rohita*, have been documented, but *Puntius sarana* has not been documented in this study, which may be due to the collection site difference. Khan and Hasan [30] conducted a study on Changhoz Dam in the nearby District Karak. They found seven species, five of which were linked to the family Cyprinidae and included *Barilius pakistanicus, Barilius vagra, Cyprinus carpio, Labeo rohita*, and *Crossocheilus latius*. Of these species, four have been documented, whereas *Crossocheilus latius*, one species, is not included in our analysis, which may be due to anthropogenic effluences or habitat destruction.

Five species such as *Salmophasia punjabensis, Amblypharyngodon mola, Schizothorax labiatus, Schizothorax esocinus*, and *Puntius chola* from the River Kurram have been reported for the first time. Because these species had already been documented from nearby rivers, their exclusion from earlier research may have been caused by differences in the period of collection, environmental conditions, climate changes, or migration of this species from other nearby freshwater bodies.

The commercially significant fish from the family Cyprinidae, including *Cyprinus carpio, Schizothorax esocinus, Schizothorax plagiostomus, Carassius auratus, Garra gotyla,* and *Tor Putitora,* are also included in the current collection. Due to unlawful fishing practices such as the use of dynamite and electric current, *Tor Putitora, Schizothorax esocinus*, and *Cyprinus carpio*, three commercial fish, are becoming increasingly scarce in these rivers. The preservation of these fish depends on effective management and conservation.

Various parameters (total hardness, temperature, copper, lead, chlorine, pH, alkalinity, iron, nitrite, and nitrate) impact the diversity of fish, according to Khan et al. [31]. The results of this investigation show that every physicochemical parameter is within the WHO's acceptable range [32].

## 5. Conclusion

The current study reported 19 species in total, such as *C. carpio, C. auratus, B. vagra, B. modestus, B. pakistanicus, S. punjabensis, A. mola, C. diplocheilus, T. putitora, L. rohita, G. gotyla, L. diplocheilus, P. sophore, P. ticto, P. conchonius, P. chola, S. plagiostomus, S. labiatus* and *S. esocinus.* 5 species (*S. punjabensis, A. mola, P. chola, S. labiatus*, and *S. esocinus*) are reported for the first time.

## Figures and Tables

**Table 1:** Classification of cyprinids collected from the Kurram River.

Order	Family	Sub Family	Genus and Species
Cypriniformes	Cyprinidae	Cyprininae	*Cyprinus carpio.*
			*Carassius auratus.*
		Danioninae	*Garra gotyla.*
			*Crossocheilus diplocheilus.*
			*Tor putitora.*
			*Labeo diplocheilus.*
			*Labeo rohita.*
		Labeoninae	*Barilius vagra.*
			*Barilius modestus.*
			*Barilius pakistanicus.*
			*Salmophasia punjabensis.*
			*Amblypharyngodon mola.*
		Barbinae	*Puntius sophore.*
			*Puntius chola.*
			*Puntius ticto.*
			*Puntius conchonius.*
			*Schizothorax labiatus.*
			*Schizothorax esocinus.*
			*Schizothorax plagiostomus.*

**Table 2:** Quantitative attributes of cyprinids at selected collection points of the Kurram River.

S. no.	Fish name	Head Works	Sukari	Jhando Kheil	Kashu Pul	Shamshi Kheil	Density	Relative density	Frequency	Relative frequency
1	*Carassius auratus*	1	2	1	2	4	2	5.31	1	1.03
2	*Cyprinus carpio*	2	2	1	2	1	1.6	4.25	1	1.03
3	*Barilius modestus*	0	1	3	1	1	1.2	3.19	0.8	0.82
4	*Barilius vagra*	3	1	2	1	2	1.8	4.78	1	1.03
5	*Barilius pakistanicus*	2	2	4	0	3	2.2	5.85	0.8	0.82
6	*Amblypharyngodon mola*	2	4	2	1	3	2.4	6.38	1	1.03
7	*Salmophasia punjabensis*	2	0	1	2	1	1.2	3.19	0.8	0.82
8	*Tor putitora*	2	1	1	2	2	1.6	4.25	1	1.03
9	*Crossocheilus diplocheilus*	0	2	1	2	1	1.2	3.19	0.8	0.82
10	*Garra gotyla*	2	3	2	1	1	1.8	4.78	1	1.03
11	*Labeo rohita*	1	2	2	1	2	1.6	4.25	1	1.03
12	*Labeo diplocheilus*	0	3	4	2	2	2.2	5.85	0.8	0.82
13	*Puntius ticto*	2	2	1	0	3	1.6	4.25	0.8	0.82
14	*Puntius sophore*	3	2	0	3	2	2	5.31	0.8	0.82
15	*Puntius chola*	0	1	2	1	1	1	2.65	0.8	0.82
16	*Puntius conchonius*	1	1	1	2	1	1.2	3.19	1	1.03
17	*Schizothorax plagiostomus*	11	8	6	5	7	7.4	19.68	1	1.03
18	*Schizothorax esocinus*	2	1	2	1	1	1.4	3.72	1	1.03
19	*Schizothorax labiatus*	2	2	1	3	3	2.2	5.85	1	1.03
Total		38	40	37	32	41	37.6	96.9		

**Table 3:** Morphometric measurements of collected cyprinids.

S. no	Fish name	T.L	S.L	F.L	H.L	E.D	Sn.L	Pre-DL	Post-D.L	Pre-PL	Post-PL	LCP	BD	BW
1	*Cyprinus carpio*	33	26	28	6.8	1.3	1.8	12.2	12.6	13	13.5	4.7	9.8	5.3
2	*Carassius auratus*	10	8.9	9	1.1	0.6	0.7	4.2	4.3	4.6	4.8	2.8	3.6	1.2
3	*Barilius vagra*	4.9	4.1	5	0.8	0.3	0.4	3	2.9	2.5	2.6	1.1	1	0.8
4	*Barilius modestus*	7	4.7	4.8	1.7	0.5	1.3	3.2	3.4	2.2	2.4	1.4	2.1	1.8
5	*Barilius pakistanicus*	8.6	6.9	7.5	1.6	0.4	0.4	5.7	5.9	6.1	6.3	1.4	1.6	1.7
6	*Salmophasia punjabensis*	12	9.2	10.1	1.8	0.3	1.7	6.8	7.2	6.7	7	1.6	2.3	0.7
7	*Amblypharyngodon mola*	4.8	4.1	3.9	0.8	0.2	0.7	1.9	1.9	1.7	1.8	0.8	1.2	0.6
8	*Crossocheilus diplocheilus*	11	9.4	10.7	2	0.4	1	6.3	6.5	6.7	6	2	2.8	2.3
9	*Tor putitora*	18	14	15.6	3.1	0.7	1.2	12.1	12	12.8	9.2	3.2	3.5	2.2
10	*Labeo diplocheilus*	16	13.2	14.3	3.2	0.4	1.7	5.8	6.9	7.5	7.4	2.6	3.9	2
11	*Labeo rohita*	15	13.2	13	2.9	1	3	10.2	10	11.1	0.4	4	5.5	3.3
12	*Garra gotyla*	18	14.5	16.4	2.9	0.4	1.6	12.2	12.3	12.6	12.4	3.2	2.9	2.6
13	*Puntius chola*	7	5	4.9	1.3	1.3	1.2	4	4.2	3.9	4.2	2.4	2	1.8
14	*Puntius conchonius*	6	5.7	4.9	1.2	0.3	1.5	3.2	3.9	4.2	4.1	2.1	1.9	1.7
15	*Puntius sophore*	7.8	6.3	6.3	1.7	0.3	1.3	5.1	4.1	4.3	4.9	1.2	2.1	1.9
16	*Puntius ticto*	9	7.8	8.9	2.3	0.4	1.2	5.9	6.9	6.9	7.9	2.3	3.9	2.8
17	*Schizothorax esocinus*	26	21	24	4.3	0.8	3.2	15.5	14.9	20	21.2	6.1	5.1	4
18	*Schizothorax plagiostomus*	21	16.7	18.5	3.7	0.9	2.3	13.5	13.8	15.4	15.3	4.3	4.6	3.1
19	*Schizothorax labiatus*	21	15.9	17.8	3.7	0.7	2.2	12.4	12.7	15.8	15.4	3.9	4.4	3.2

Abbreviations: BD = body depth; BW = body width; E.D = eye diameter; F.L = forked length; H.L = head length; LCP = length of caudal peduncle; Post-D.L = postdorsal length; Post-PL = postpelvic length; Pre-DL = predorsal length; Pre-PL = prepelvic length; S.L = standard length; Sn.L = snout length; T.L = total length.

**Table 4:** Physicochemical parameters (mg/L) of Kurram River.

Parameters	Head Works		Sukari		Jhando Kheil		Kashu Pul		Shamshi Kheil		WHO limit
	Minimum	Maximum	Minimum	Maximum	Minimum	Maximum	Minimum	Maximum	Minimum	Maximum	
Temperature (°C)	17 ± 1.00	21 ± 1.00	17.8 ± 1.00	22 ± 1.00	18 ± 1.00	22 ± 1.00	18.5 ± 1.00	**22.8 ± 1.00**	19 ± 1.00	22 ± 1.00	16C–40°C
Alkalinity (mg/L)	78 ± 2.08	129 ± 4.58	82 ± 2.08	134 ± 4.58	68 ± 2.08	130 ± 4.58	89 ± 2.08	**145 ± 4.58**	88 ± 2.08	**145 ± 4.58**	10–400 mg/L
Total Hardness(mg/L)	65 ± 5.00	129 ± 3.79	68 ± 5.00	132 ± 3.79	70 ± 5.00	124 ± 3.79	72 ± 5.00	**150 ± 3.79**	68 ± 5.00	139 ± 3.79	10–400 mg/L
Nitrate (mg/L)	3.6 ± 1.62	08 ± 1.42	3.3 ± 1.62	08 ± 1.42	2.4 ± 1.62	**09 ± 1.42**	2.2 ± 1.62	08 ± 1.42	2.4 ± 1.62	08 ± 1.42	45 mg/L
Copper (mg/L)	0.05 ± 0.004	0.08 ± 0.13	0.03 ± 0.004	0.07 ± 0.13	0.04 ± 0.004	0.09 ± 0.13	0.03 ± 0.004	**0.1 ± 0.13**	0.04 ± 0.004	**0.1 ± 0.13**	2 mg/L
Iron (mg/L)	0.02 ± 0.0015	0.03 ± 0.0019	0.01 ± 0.0015	**0.03 ± 0.0019**	0.01 ± 0.0015	**0.03 ± 0.0019**	0.02 ± 0.0015	0.003 ± 0.0019	0.001 ± 0.0015	0.002 ± 0.0019	0.3 mg/L
Nitrite (mg/L)	0.01 ± 0.02	0.07 ± 0.01	0.01 ± 0.02	0.07 ± 0.01	0.01 ± 0.02	0.05 ± 0.01	0.02 ± 0.02	**0.08 ± 0.01**	0.02 ± 0.02	**0.08 ± 0.01**	0.1 mg/L
Chlorine (mg/L)	0.1 ± 0.09	**0.9 ± 0.10**	0.1 ± 0.09	**0.9 ± 0.10**	0.1 ± 0.09	0.8 ± 0.10	0.09 ± 0.09	0.7 ± 0.10	0.08 ± 0.5	0.5 ± 0.10	5 mg/L
Lead (mg/L)	0.001 ± 0.001	0.003 ± 0.001	0.001 ± 0.001	**0.005 ± 0.001**	0.001 ± 0.001	**0.005 ± 0.001**	0.002 ± 0.001	0.004 ± 0.001	0.001 ± 0.001	0.002 ± 0.001	0.01 mg/L
Ph	6.8.0 ± 0.10	7.9 ± 0.10	6.9 ± 0.10	**8.0 ± 0.10**	6.8 ± 0.10	7.9 ± 0.10	7.0 ± 0.10	7.7 ± 0.10	7.1 ± 0.10	7.8 ± 0.10	6.5–8.5

*Note:* Bold figures show the maximum mean values.

**Table 5:** Diversity indices of the family Cyprinidae in the River Kurram, Bannu.

Diversity indices	Head Works	Sukari	Jhando Kheil	Kashu Pul	Shamshi Kheil
Individuals	38	40	37	32	41
Dominance_D	0.1233	0.085	0.07962	0.07617	0.07674
Simpson_1-D	0.8767	0.915	0.9204	0.9238	0.9233
Shannon_H	2.442	2.692	2.707	2.705	2.755
Evenness_e^ H/S	0.7661	0.8203	0.8322	0.8797	0.8278
Margalef	3.849	4.608	4.708	4.617	4.847

**Table 6:** Factor loading of the family Cyprinidae in the River Kurram, Bannu.

	F1	F2	F3	F4	F5
Head works	0.69117	−0.39914	−0.57869	−0.077029	−0.14882
Sukari	0.46965	0.26838	0.52224	0.14338	−0.64351
Jhando Kheil	0.29155	0.76359	−0.31304	0.33248	0.35127
Kashu Pul	0.23275	−0.42583	0.37093	0.65828	0.43997
Shamshi Kheil	0.40315	0.065282	0.39598	−0.65546	0.49677

## Data Availability

Data are available on reasonable request from the authors.
